# Holographic lasing with dielectric metasurfaces

**DOI:** 10.1126/sciadv.aea7345

**Published:** 2026-05-29

**Authors:** Ayesheh Bashiri, Aleksandr Vaskin, Muyi Yang, Katsuya Tanaka, Marijn Rikers, Thomas Pertsch, Isabelle Staude

**Affiliations:** ^1^ Institute of Solid-State Physics, Friedrich Schiller University, Jena, 07743, Germany.; ^2^ Abbe Center of Photonics, Institute of Applied Physics, Friedrich Schiller University, Jena, 07745, Germany.; ^3^ Max Planck School of Photonics, Jena, 07745, Germany.; ^4^ Department of Quantum Science and Technology, Research School of Physics, Australian National University, Canberra, ACT 2601, Australia.; ^5^ Fraunhofer-Institute of Applied Optics and Precision Engineering IOF, Jena, 07745, Germany.

## Abstract

Light-emitting metasurfaces provide a compact, integrated solution for simultaneous light generation and beam shaping, making them a promising candidate for advanced photonic applications. However, existing approaches for tailoring far-field emission patterns either operate in the spontaneous emission regime, where low coherence limits precise light control, or rely on passive holographic metasurfaces that shape externally supplied coherent (or partially coherent) illumination. Here, we present a light-emitting metasurface system with holographic lasing output, composed of a binary-structured metasurface integrated with a gain medium, that enables coherent light generation and precise beam shaping of the output lasing emission within a single device. Through our design approach, the device sustains robust lasing performance despite structural disorder introduced for holographic encoding. With a compact ultraflat footprint, low lasing threshold, and wide field of view, our system offers an exceptional platform for generating design-specified structured lasing emission, with notable potential for miniaturized optical systems.

## Teaser

Dielectric metasurfaces enable lasing emission and pixel-level beam shaping in a single device.

## INTRODUCTION

Developing an ultracompact laser source with built-in spatial wavefront shaping remains a major challenge in nanophotonics, with substantial implications for augmented reality (AR), light field displays, deep diffractive neural networks, and next-generation medical diagnostics and treatment. Metasurfaces, planar optical elements enabling wavefront control, have emerged as promising tools in this context (*1*–*4*). When combined with active materials, light-emitting metasurfaces can simultaneously generate and shape light (*5*–*8*); however, operating in the spontaneous emission regime, they suffer from limited temporal and spatial coherence, restricting their ability to form interference-based complex patterns.

In contrast, operating in the lasing regime establishes coherence among emitted photons, producing light with well-defined phase relationships and enabling precise wavefront shaping for holographic applications. Lasing metasurfaces, which integrate resonant nanostructures with optical gain, have recently emerged as compact, coherent light sources (*9*–*12*). However, most remain restricted to simple, fixed-direction beams, lacking the capability to project complex spatial patterns, with few exceptions.

One approach to overcoming this limitation is to integrate metasurface reflectors into external cavity lasers as the beam shaping element (*13*). However, they remain limited by reliance on external laser diodes, precise alignment, and bulky configurations. Another strategy uses metasurface-integrated vertical cavity surface emitting lasers (VCSELs), with dielectric metasurfaces monolithically fabricated atop VCSEL apertures for directional beam shaping (*14*, *15*). While offering better integration, these devices face challenges such as complex epitaxial growth and cavity thickness exceeding tens of microns. Moreover, placing the metasurface outside the cavity limits its function to postemission beam shaping. In contrast, embedding the metasurface within the laser cavity additionally allows for direct control over lasing threshold, wavelength, and polarization. This approach enhances light-matter interaction and local density of optical states (LDOS), boosting the Purcell factor and spontaneous emission coupling ratio, enabling low-threshold, multifunctional lasing in compact, subwavelength architectures. Yet, simultaneous generation and arbitrary shaping of lasing emission within a single metasurface remains largely unexplored. Very recently, Zeng *et al.* (*16*) demonstrated a quasi-bound state in the continuum (q-BIC) metasurface laser that generates polarization-dependent holographic patterns via geometric phase, implemented by rotating an off-center aperture in each Si_3_N_4_ meta-atom. While the work marks an important advance in metasurface-based lasers, it does suffer from several practical limitations. The demonstrated angular control of the lasing beam is limited to ≈ ± 10°. In addition, the design requires precise per-pixel hole rotation to encode phase, and the hologram is visible only in the intended circular polarization, restricting practical use unless additional polarization optics are placed after the device. Moreover, maintaining a high-quality factor (high-*Q*) in such q-BIC designs requires nanometer-scale precision in aperture size and rotation, making the performance sensitive to fabrication tolerances.

**Fig. 1.**
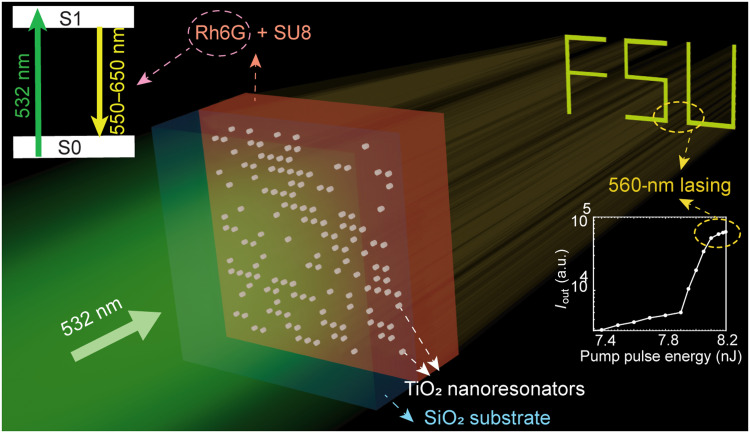
Schematic illustration of on-off binary lasing metasurface. An engineered array of TiO_2_ nanoresonators on a silicon dioxide (SiO_2_) substrate is integrated with an SU8 layer containing Rh6G laser dye. Under 532-nm excitation, the coupled system generates lasing at 560 nm with a designed far-field emission pattern. a.u., arbitrary units.

Here, we report a binary holographic metasurface laser that unites coherent light generation and pixel-level spatial control in a single compact device (Fig. 1). The system consists of a titanium dioxide (TiO_2_) dielectric metasurface, spin-coated with a SU8 resist layer doped with Rhodamine 6G (Rh6G) laser dye as the gain medium. The latter is chosen for its high quantum yield, broad gain spectrum, and ease of integration. Our design uses a binary “on-off” metasurface configuration, wherein spatial modulation of the emission is encoded by the presence (on) or absence (off) of nanoresonators across the array. This enables programmable holographic pattern projection via engineered intracavity feedback. With a compact, ultraflat footprint (≈37 μm by 37 μm lateral area, <1-μm thickness), a relatively low lasing threshold of <8 nJ pump pulse energy (0.58 mJ/cm^2^), and the ability to project patterns over a high field of view (FOV) of 100° × 100°, the device demonstrates exceptional performance for generating structured light. As a proof of concept, we have successfully projected the abbreviation “FSU” in the holographic output, representing “Friedrich-Schiller-University.” This work advances the integration of holography and lasing within a single, ultraflat platform, bridging the gap between passive metasurfaces and conventional laser systems through a robust, polarization-independent design. This paves the way for structured-light applications, such as AR contact lenses, where each device functions as both light emitter and projector, for nonlinear hidden layers of diffractive neural networks exploiting the inherent optical nonlinearities associated with lasing action, or for light field displays, where individual metasurface lasers could serve as scalable, self-contained pixel-microlens units.

Our approach relies on second-order distributed feedback and guided mode resonance to achieve low-threshold lasing. The periodic high-index nanoresonator array forms a photonic bandgap for laterally guided modes in the SU8 layer by fulfilling the second-order Bragg diffraction condition at the design wavelength, thereby enabling in-plane optical feedback essential for lasing (*9*). In addition to coupling to guided modes, the resonant scatterers support collective surface-lattice resonance (SLR)–like interactions (*17*), further enhancing feedback through diffractive coupling. Near the photonic band edge, the dispersion flattens, giving rise to a slow-light regime with enhanced LDOS and stronger light-matter interaction within the gain medium. In addition, flat dispersion leads to a reduced mode volume, as the spatial extent of the mode corresponds to the Fourier transform (FT) of its angular emission spectrum. When the mode volume becomes smaller than the physical dimensions of the metasurface, edge truncation no longer introduces strong scattering losses. As a result, radiative losses are suppressed, and the mode maintains a high-*Q* factor even for a finite metasurface size (*18*–*20*). Using low-loss dielectric materials minimizes dissipation, further enhancing the *Q* factor and feedback efficiency. Second-order Bragg diffraction additionally enables vertical outcoupling of the guided modes, supporting lasing emission normal to the surface. However, by spatially patterning the metasurface—through the binary presence or absence of nanoresonators—this emission is redistributed into a broad angular range, allowing structured light projection over a wide FOV.

## RESULTS

### Design and fabrication

The design of our lasing metasurface was performed in two steps: First, we designed the periodic lasing metasurface; second, we used an inverse-design approach to determining the spatial arrangement of nanoresonators that encodes the target far-field hologram. These steps are described in detail in the following sections.

In the first step, using the finite element method (FEM) implemented by the software package COMSOL Multiphysics, we designed a periodic metasurface composed of a two-dimensional (2D) square array of resonant TiO_2_ (see fig. S1 for refractive indices) nanocylinders on a silicon dioxide (SiO_2_) substrate and an SU8 photoresist coating acting as a 2D planar waveguide. The optical confinement along the out-of-plane direction leads to a strong in-plane amplification of spontaneous emission in favor of lasing. Note that the Rh6G laser dye was not considered explicitly in numerical simulations; in the experiments, the dye was mixed into the SU8 film before spin coating the film onto the metasurface.

In the design process, we jointly optimized the nanocylinder geometry, array period, and SU8 film thickness to realize a high-*Q* band edge mode overlapping with the Rh6G emission band and providing strong field enhancement within the gain medium. These two criteria are intrinsically coupled as the same resonance with maximized *Q* at Γ point also concentrates the optical field in the SU8 layer, ensuring efficient light-matter interaction. In addition, the design was tailored to support the formation of a stop band at Γ, which strengthens in-plane distributed feedback and preserves high-*Q* operation even in the reduced-size array. The presence of the stop band and the associated flattening of the dispersion near the band edges decreases the lasing mode volume and minimizes edge-related radiative losses when the metasurface size is reduced, thereby facilitating efficient coherent emission from compact devices.

The array period is chosen to satisfy the second-order Bragg diffraction condition, providing in-plane optical feedback. Simultaneously, the nanocylinders are designed to support Mie-type resonances (*21*–*23*) at the target wavelength. Together, the periodicity and resonator response enable collective SLRs, which enhance scattering strength, promote bandgap formation, and improve optical feedback. Our simulations assumed periodic boundary conditions to model infinitely periodic metasurfaces and did not account for the finite size and perturbed periodicity of the metasurfaces. A detailed optimization workflow is described in Materials and Methods. The optimized metasurface parameters are listed in table S1.

**Fig. 2.**
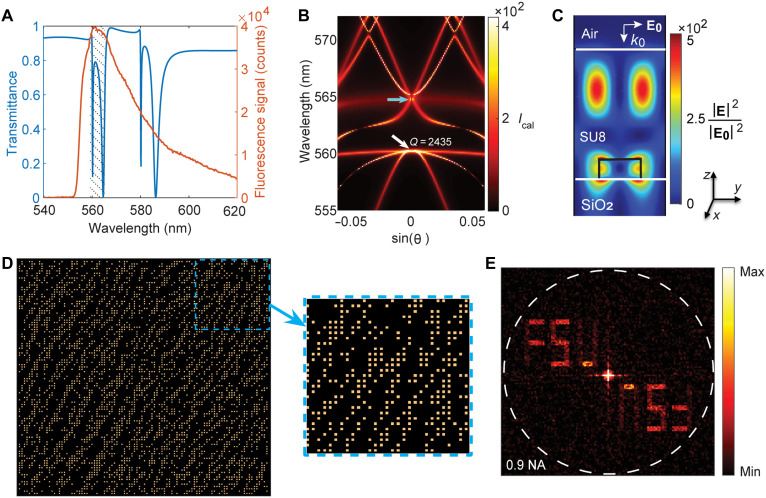
The two-step design process of on-off binary lasing metasurface. First step: (**A**) Numerically calculated linear optical transmission spectrum of the designed metasurface coated with SU8 layer for y-polarized normal incidence illumination (blue) and measured fluorescence spectrum of an SU8 film of the same thickness containing Rh6G on a bare glass substrate (red) using a 550-nm band-pass filter. The range of interest is indicated with dashed hatching. (**B**) Simulated angle- and wavelength-resolved emission enhancement by the metasurface for azimuthal angle φ = 90° and polar angle θ up to ±3° averaged over TE and TM polarization of the incident light. The white and blue arrows indicate the lower and upper edges of the stop band, respectively. The resonance *Q* factor at 560 nm and θ = 0 is 2435. *I*_cal_ stands for electric field intensity enhancement with respect to incidence plane wave illumination, defined as *I*_cal_ = $\langle \frac{{|E|}^{2}}{{|E_{0}|}^{2}}\rangle$. Here, the average is taken over the SU8 layer. (**C**) Calculated near-field intensity profiles at 560 nm in the vertical *y*-*z* cross section through the center of the unit cell for normal incidence illumination, normalized with respect to the intensity of the incident plane wave. The outlines of the nanoresonator are shown in black. Second step: (**D**) Optimized arrangement of the nanoresonators in the binary metasurface, which generates an output FT forming the letters FSU. (**E**) The absolute value of the discrete FT of the optimized array in part (D).

Figure 2A shows the calculated transmission spectrum of the designed metasurface coated with an SU8 resist, under normal incidence plane wave illumination, together with the measured emission spectrum of a SU8 film of the same thickness containing Rh6G on a bare substrate. The results indicate that the designed metasurface supports high-*Q*, spectrally sharp resonances that lie within the Rh6G emission band.

Figure 2B shows the simulated angle- and wavelength-resolved emission enhancement of the dipole emitters coupled to the designed metasurface (relative to the emission of the same emitters in vacuum), obtained via the reciprocity principle (see Materials and Methods) (*5*). We observe multiple bands corresponding to the diffractive coupling to the guided modes of the SU8 layer and lattice modes of the metasurface. For a metasurface with a period of 365 nm, the lasing high-*Q* mode, indicated by a white arrow, appears at a wavelength of 560 nm, which is spectrally adjacent to the emission peak of Rh6G and satisfies the second-order Bragg diffraction condition at a waveguide mode index of *n*_wg_ = 1.53 (*9*). We identify a stop band between 560 and 565 nm (white and blue arrows in Fig. 2B) at the polar angle θ = 0 (Γ point) with a gap-midgap ratio of $\frac{\Delta \omega }{\omega }$ of ≈0.9%, where ω and Δω represent the central angular frequency and the stop band width, respectively. The stop band width serves as a direct measure of the Bragg length, which corresponds to the number of lattice planes required for 1/*e* diffraction efficiency (*9*, *24*–*26*). In particular, the inverse of the relative stop band width (0.9%) provides an estimate for the number of unit cells required for the Bragg diffraction (≈100 × 100) in the periodic system. This approach allowed us to minimize the metasurface footprint while ensuring low lateral losses, thereby maintaining a high-*Q* factor essential for lasing. The calculated *Q* factor at the lower band edge (560 nm) is ≈2.4 × 10^3^, which is sufficiently high to support lasing (*27*–*29*). We also evaluated the *Q* factors at two additional angles along the same dispersive band (see fig. S2B), confirming that the highest *Q* occurs near the band edge where the dispersion is flat. Details are provided in Supplementary Text.

Figure 2C shows the normalized electric field intensity distribution in the vertical *y*-*z* cross section through the center of the unit cell for normal incidence at the resonant wavelength. Notably, the designed structure achieves a strong electric field intensity enhancement of more than two orders of magnitude in the active medium. Together, these results demonstrate that the designed metasurface meets all key performance objectives.

Generating programmable emission patterns with metasurfaces typically requires aperiodic arrangements and/or in-plane variations in the shape and size of the constituent nanoresonators (*30*–*33*). To this end, in the second design step, we used inverse design using gradient-free global genetic optimization (*34*) to create an on-off binary metasurface by selectively removing or retaining nanoresonators. The optimization continued until the metasurface produced the desired far-field emission pattern FSU, as the abbreviation of Friedrich-Schiller-University.

The far-field pattern generated by the binary metasurface is determined by the convolution of the lasing mode angular emission profile with the FT of the real-space binary metasurface array (*30*, *31*). Here, the lasing mode is outcoupled via scattering from the nanoresonators, which act as secondary sources. The light from these secondary sources interferes, forming the far-field emission pattern, with their relative phases determined by the in-plane wave vector of the lasing mode (*k*_||_ = 0) in our system. Assuming negligible distortions from metasurface truncation to 100 × 100 unit cells and periodicity perturbations, the secondary sources can be considered to all have the same scattering strength. Consequently, the far-field pattern is entirely governed by the spatial arrangement of the nanoresonators in the binary metasurface and the phase distribution imposed by the lasing mode, with the interference in the far-field representing the FT in the source plane (*31*, *35*). Further details of the optimization process are provided in Materials and Methods and fig. S3.

Figure 2D illustrates the optimized spatial arrangement of the binary metasurface, where bright and dark pixels represent the presence or absence of nanoresonators, respectively. The metasurface measures ~37 μm by 37 μm, consisting of 100 × 100 unit cells (each 365 nm in size), with ~36% of the nanoresonators removed. Figure 2E shows the absolute value of the discrete FT of the optimized array, which defines the far-field emission pattern of the binary lasing metasurface, revealing a central peak that corresponds to the initial periodic arrangement and two symmetrically arranged FSU in the second and fourth quarters. This symmetry arises from the inherent properties of FT.

**Fig. 3.**
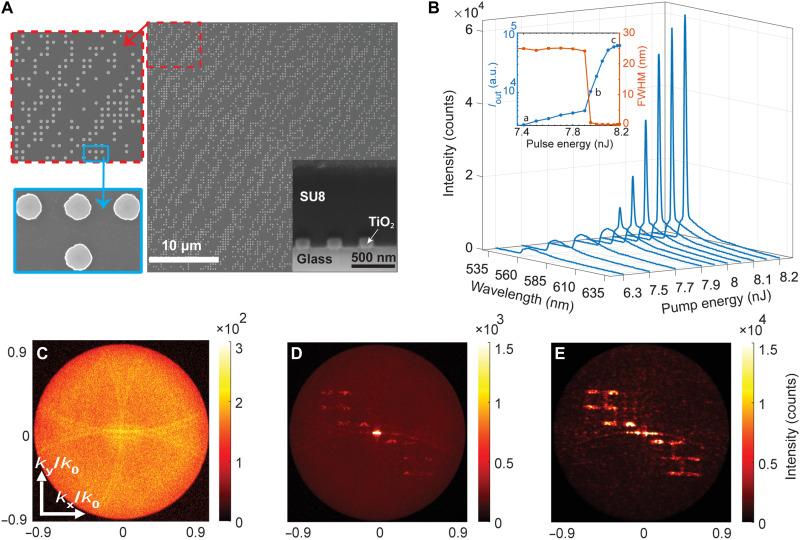
On-off binary lasing metasurface enabling coherent light generation with precise spatial control of the output beam. (**A**) The top-view SEM image of a typical fabricated metasurface with a period of 365 nm. The inset shows a FIB cross section of the metasurface coated with 880-nm SU8 film doped with Rh6G laser dyes. (**B**) Evolution of the emission spectra of the same metasurface at different pump pulse energies. The inset represents the maximum output intensity *I*_out_ (blue curve) and FWHM of the emission peak (red curve) at different pump energies, revealing three regimes: (i) spontaneous emission dominated regime, (ii) onset of lasing near the threshold (~7.95 nJ, 0.581 mJ/cm^2^), and (iii) gain saturation at higher pump powers. (**C** to **E**) Measured BFP image of the lasing metasurface using a 560-nm band-pass filter: (C) Below threshold (7.8 nJ), it shows the diffraction bands of the spontaneous emission. (D) Right above threshold (8 nJ), the onset of lasing emission manifests as a bright, narrow feature in the center, accompanied by the appearance of the target FSU pattern. (E) Well past the threshold (8.8 nJ) and after spatially filtering the strong central peak, the target FSU pattern becomes prominent.

We fabricated (see Materials and Methods) a set of metasurfaces with varying design parameters to account for fabrication imperfections. The fabricated metasurfaces were spin-coated with SU8 photoresist mixed with 0.165 wt % Rh6G dye (see Materials and Methods). A top-view scanning electron microscope (SEM) image of a typical fabricated binary metasurface and a focused ion beam (FIB) cross-sectional SEM image of the metasurface after spin coating are depicted in Fig. 3A and its inset, respectively. The spin-coated SU8 film appears uniform and has a thickness of *h*_wg_ = 880 nm. The dimensions of the fabricated metasurface, listed in table S1, closely match the optimized parameters from the design step.

### Optical characterization and lasing performance

Next, we experimentally characterized the fabricated metasurfaces. We pumped the sample using a 532-nm pulsed laser (Teem Photonics, model STG-03E-130) with a pulse width of 0.5 ns and a maximum pulse energy of 3 μJ. Unless otherwise specified, all measurements in our work were conducted at a repetition rate of 1 Hz, with each acquisition integrating over a 1-s period. We performed power-dependent fluorescence emission spectroscopy (see Materials and Methods and fig. S4 for details on the experimental setup). The results, shown in Fig. 3B, present the output emission intensity as a function of wavelength and pump pulse energy for the spin-coated metasurface. At a pump pulse energy below 7.9 nJ, we observe a broad spectrum featuring the spontaneous emission from Rh6G dye. Upon increasing the pump pulse energy to 7.95 nJ (0.581 mJ/cm^2^) and beyond, we observe a narrow peak emerging at a wavelength of 560 nm, corresponding to the lasing mode in this system. The inset of Fig. 3B presents output intensity (blue) and the full width at half maximum (FWHM) (red) as functions of pump pulse energy. The output intensity exhibits the characteristic S-curve, revealing three distinct regimes: (i) spontaneous emission-dominated regime at lower pump energies; (ii) the transition to amplification and onset of lasing starting at ≈7.95 nJ, characterized by a pronounced increase in slope; and (iii) gain saturation at higher excitation levels. The corresponding FWHM decreases sharply above threshold, indicating pronounced spectral narrowing consistent with the onset of optical feedback within the cavity. Linewidth extraction accounts for the finite spectrometer resolution and removal of the broad fluorescence background (see Supplementary Text for details) and results in a minimum of ≈0.26 at 560 nm, corresponding to a quality factor of *Q* ≈ 2.2 × 10^3^.

Further, we performed back focal plane (BFP) imaging of emission (Materials and Methods) to investigate the evolution of the far-field radiation pattern of the metasurface from below to above the lasing threshold. All BFP images are measured using a 560-nm (10-nm bandwidth) band-pass filter in the detection path. Figure 3C demonstrates the BFP image below the threshold at 7.8 nJ in the spontaneous emission regime. The main features in this regime are the diffraction modes of the structure, which originate from the periodic arrangement of the nanoresonators. Note the convergence of distinct diffraction orders at the normal direction (center of the BFP image), which is consistent with the formation of SLRs. As the pump pulse energy increases to 8 nJ, the system approaches the lasing threshold. In the corresponding BFP image (Fig. 3D), this manifests as weakening of the diffractive features of the spontaneous emission and the simultaneous emergence of a sharp peak at the center of the BFP image (*k*_x_, *k*_y_ = 0), where *k*_x_ and *k*_y_ are the in-plane components of the photon momentum. This highly directive peak indicates a coherent lasing emission and is accompanied by a subtle formation of our target pattern FSU, already at this pump pulse energy. Last, at a pump pulse energy well above the threshold (8.8 nJ) and after spatially filtering the strong central lasing peak for better visibility, we can observe (Fig. 3E) the target FSU pattern, generated by our binary lasing metasurface and showing a very good agreement with the simulated pattern in Fig. 2E.

To compare the quality of the holographic image predicted by design and obtained from the experimental result, we quantitatively analyzed hologram efficiency, defined as $\eta =\frac{I_{\text{target}}}{I_{\text{total}}}$, where $I_{\text{target}}$ is the integrated intensity within the projected FSU pattern and $I_{\text{total}}$ is the total transmitted intensity, above threshold. We perform this analysis on the BFP image shown in Fig. 3D with the zeroth-order lasing peak present and the computed FT pattern shown in Fig. 2E. Both the measured BFP image and computed far-field pattern exhibit similar efficiencies of ~17%, indicating strong agreement between experiment and simulation.

The relatively low efficiency primarily arises from the dominant zeroth-order lasing emission, which contributes most of the total output intensity and is intrinsically coupled to hologram formation in this simple binary intracavity design. The holographic image appears and disappears together with the central lasing peak, confirming strong dependence on the zeroth-order mode. This behavior, anticipated in simulation and reproduced experimentally, validates the device as a proof-of-concept demonstration of lasing holography based on a binary-structured metasurface. We further evaluated the intensity uniformity of the holographic pattern following the method in (*33*) (see Supplementary Text) and found a close agreement between the theory and experiment.

**Fig. 4.**
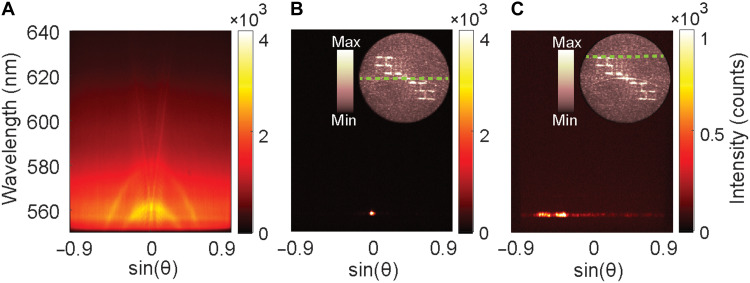
Momentum-resolved spectroscopy further confirms that the on-off binary metasurface enables complete directional control of the self-generated lasing emission. (**A**) A thin slice (*k*_y_ = 0) of the BFP image shown in Fig. 3C is spectrally resolved, showing the band diagram of the metasurface below the threshold. Note that for better visibility, the measurement below the threshold is performed with an excitation laser repetition rate of 4 kHz and integrated for 30 s. (**B**) Above the threshold, the emission is fully concentrated at the particular point of (θ = 0, λ = 560 nm). Similar to (A), as shown in the inset with a green dashed line, the slice is cut from the center of the BFP image (*k*_y_ = 0). (**C**) Well above the threshold, where the target pattern emerges clearly, a cross section through the upper part of the generated pattern (see inset) is spectrally resolved, revealing the lasing emission directed toward oblique angles at 560 nm.

To further characterize the lasing emission, we performed momentum-resolved spectroscopy measurements by spectrally resolving a thin slice of the respective BFP images (Materials and Methods) around *k*_y_ = 0. Figure 4A illustrates the results below the lasing threshold. Similar to the calculated wavelength- and angle-resolved emission enhancement shown in Fig. 2B (covering a limited range of λ and θ with fine resolution) and fig. S5 (spanning a broader range of λ and θ with coarser steps for qualitative comparison), we observe highly dispersive narrow bands corresponding to the guided modes in the metasurface slab. However, because of the perturbed periodicity of the metasurface, the limited resolution of our imaging spectrometer, and noise, fine features are not thoroughly resolved in the measured data. Nevertheless, we can identify the origin of the lasing mode at (θ = 0, λ = 560 nm).

Above threshold (Fig. 4B), the emission is concentrated at a single point of (θ = 0, λ = 560 nm), demonstrating the high degree of spatial and temporal coherence of the lasing emission. The inset in Fig. 4B illustrates the *k*_y_ = 0 cross section of the corresponding BFP image (Fig. 3D) with a green dashed line. Figure 4C illustrates the same measurement repeated for the cross section through the FSU pattern (inset of Fig. 4C). As we expected, the lasing emission appears as multiple dots constructing the top lines of the displayed letters, spectrally narrowed down to the same wavelength of 560 nm but directed to the design angles. Thus, this binary metasurface shows great opportunities for spatially tailoring the self-generated lasing emission.

Furthermore, we examined an additional fabricated metasurface (Supplementary Text and figs. S6 to S8), featuring the same spatial distribution of nanoresonators but slightly different nanoresonator dimensions, which supports multimode lasing action. The observed doubling of the target image (figs. S7 and S8) further underpins the rich engineering options offered by the combined effect of lasing mode and structure (array) factor on far-field shaping. We discuss the feasibility and experimental challenges associated with second-order photon-correlation measurements in our dye-based metasurface laser in Supplementary Text.

It is important to note that metasurfaces can also generate holographic images from nonlasing emission. For instance, photoluminescence (PL) coupled into a planar waveguide mode can provide an effective illumination source with enhanced spatial coherence, while the on-chip metasurface passively modulates and out-couples the guided PL, encoding the target wavefront (*36*). Similarly, partially coherent amplified spontaneous emission used as external illumination can be shaped by a metasurface into holographic images (*37*). However, our device both generates and shapes the lasing emission, with the lasing action verified by clear threshold behavior, pronounced spectral narrowing to a single band edge mode, and distinct *k*-space selectivity associated with the designed cavity feedback.

## DISCUSSION

We experimentally demonstrated a binary holographic metasurface laser that combines coherent light generation with pixel-level beam shaping in a single compact device. The metasurface laser projects holograms over a 100° × 100° FOV and enables programmable far-field pattern formation, as exemplified by the FSU output. By leveraging second-order distributed feedback and guided mode resonance, the metasurface sustains a high-*Q* band edge mode that remains operational even when numerous unit cells are intentionally removed to encode the hologram, demonstrating robustness to fabrication imperfections and structural disorder. Unlike typical quasi-BIC lasers, whose ultrahigh-*Q* modes are easily degraded by such perturbations, our design maintains stable lasing with a relatively low threshold (<8 nJ per pulse, 0.58 mJ/cm^2^).

A major advantage of this platform is its intrinsic compactness, with gain, feedback, and holographic projection fully integrated within an ultraflat, compact footprint structure (≈37 μm by 37 μm lateral area, <1-μm thickness), eliminating the need for external beam shaping or collimation components. Beyond the proof-of-concept demonstration, our approach opens opportunities for integrated light field displays, optical interconnects, light detection and ranging (LiDAR), and on-chip photonic computing systems where lasing and beam shaping are intrinsically merged within a flat optical platform.

The measured holographic diffraction efficiency reaches about 17%, in excellent agreement with the design and confirming accurate experimental reproduction of the predicted pattern, although the efficiency remains relatively low due to dominant zeroth-order lasing peak. In addition, the projected pattern appears in the second quadrant of the BFP image and is accompanied by its mirror in the fourth quadrant, a consequence of the inherent symmetry in the FT of real-valued holograms. To address these limitations and looking ahead, applying more sophisticated optimization techniques such as gradient-based inverse design aiming at suppression of the central emission peak, as well as designing meta-atoms with both phase and amplitude control (*38*–*40*), will substantially improve hologram formation efficiency in future works. In parallel, adopting more photostable gain media such as III-V semiconductor-based platforms (*41*) and advancing toward electrical pumping would enhance device reliability and integration, ultimately enabling fully programmable flat lasers that combine high-*Q* performance, compactness, and precise spatial control.

## MATERIALS AND METHODS

### Metasurface design

We designed the periodic metasurfaces using the FEM implemented in COMSOL Multiphysics. In simulations, the metasurface was illuminated by a normal incident plane wave, and periodic boundary conditions were applied to restrict the computational domain to a single unit cell.

The lattice period was chosen so that the guided mode near 560 nm satisfies the second-order Bragg diffraction condition, providing in-plane distributed feedback via the coupling of counter-propagating waves. This opens a stop band at the Γ point and provides a vertical radiation channel at normal incidence. For a period of 365 nm, the effective mode index is *n*_wg_ = 1.53, satisfying the Bragg condition and positioning the band edge mode at the target wavelength. The nanocylinder dimensions were optimized to align a Mie-type resonance with the target wavelength, enhancing diffractive coupling and promoting strong lattice-mediated interactions (SLR-like behavior). The SU8 thickness was tuned to support a single guided mode, suppressing higher orders. This ensures strong spatial overlap between the resonant field and the gain medium while avoiding mode competition. Considering all these and after defining the reasonable range for period and SU8 thickness, we performed a joint parametric sweep over all the parameters to maximize the volume-averaged ∣**E**∣^2^ in SU8 in a wavelength range between 560 and 590 nm, spectrally overlapping with the emission peak of Rh6G dyes.

### Calculation of the angle- and wavelength-resolved emission enhancement below threshold

We simulated the angle- and wavelength-resolved emission enhancement from the metasurface coated with an SU8 film containing randomly oriented and homogeneously distributed point dipole emitters in COMSOL Multiphysics based on the reciprocity principle (*5*). Using this method, the computational domain was reduced to a single elementary unit cell with periodic boundary conditions while accounting for the isotropic orientation of the emitting dipoles. Specifically, we consider a dipole **p**_1_ located at position **r**_1_ on the periodic metasurface, representing the emitter of interest, and a second dipole **p**_2_ positioned in the far-field (**r**_2_) along a given observation direction represented by polar angle θ and azimuthal angle φ. In this configuration, the reciprocity principle states that **p**_2_ **· E**_1_(**r**_2_) = **p**_1_ **· E**_2_(**r**_1_), where **E**_1_(**r**_2_) corresponds to the far-field radiation emitted by **p**_1_ into the direction (θ,φ) through its coupling to the metasurface, and the field **E**_2_(**r**_1_) represents the local electric field at the position **r**_1_ (where **p**_1_ is located) and induced by a plane wave incident from the same direction (θ,φ) generated by the far-field source **p**_2_. The polarization of this reciprocal plane wave [transverse electric (TE) or transverse magnetic (TM)] is determined by the orientation of the far-field dipole **p**_2_. As a result, the directional emission of a dipole into (θ,φ) can be obtained by evaluating the local field at **r**_1_ under reciprocal plane wave illumination, without explicitly simulating a dipole source in a periodic structure.

Accordingly, the power *P*(θ,φ;r) emitted by a randomly fluctuating dipole **p** located at **r** into the far-field direction (θ,φ) is proportional to the reciprocity-derived local field intensity ${|E_{\text{local}}\left(\theta ,\varphi ;r\right)|}^{2}$. Here, $E_{\text{local}}\left(\theta ,\varphi ;r\right)$ denotes the local electric field at **r** induced by a TE- or TM-polarized plane wave incident from a direction (θ,φ) at the wavelength corresponding to the ED transition.

After averaging over isotropically oriented emitters, the emission intensity into the direction (θ,φ) is calculated as *P*(θ,φ) $\propto \langle \frac{\sum_{\text{TE},\text{TM}}{|E_{\text{local}}\left(\theta ,\varphi ;r\right)|}^{2}}{E_{0}^{2}}\rangle$, where *P*(θ,φ) is the emitted power at the wavelength of interest, $E_{0}$ is the amplitude of the incident plane wave electric field, and ⟨·⟩ denotes spatial averaging over the active volume containing the emitters **p**.

### Genetic algorithm optimization process for metasurface design

The metasurface comprises a 100 × 100 array of nanoresonators arranged in a periodic lattice, encoded by a binary vector of 10,000 elements. Each element was assigned a value of 1 or 0, indicating the presence or absence of a nanoresonator, respectively. To optimize these vectors, a genetic algorithm (*33*, *34*) was used, iteratively refining the configuration. The far-field emission pattern for a given vector was computed by applying the discrete FT to the nanoresonator arrangement. This pattern was then compared to the target pattern, the letters FSU. For both the simulations and the target, a selected region of *k*-space containing the FSU symbol was normalized, and the mean squared error was calculated. The optimization aimed to minimize this error, ensuring the desired pattern was accurately reproduced. A flowchart outlining the overall process is shown in fig. S3.

### Device fabrication

The fabrication of the metasurfaces was carried out using electron beam lithography (EBL) in combination with an atomic layer deposition (ALD)–based planarization process to achieve straight sidewalls for the nanodisks. First, a 220-nm-thick photoresist layer (AZ 1505) was spin-coated onto the SiO_2_ substrate as a support layer for the TiO_2_ structure. To enhance surface conductivity, a 15-nm chromium (Cr) layer was deposited via ion beam deposition (Oxford Instruments, Ionfab 300). Next, an electron beam resist (AR-P 6200.04) was spin-coated and exposed by a variable-shaped EBL system (Vistec SB350). This thin electron beam resist serves as the pattern-defining layer and is used since it is fully compatible with our established exposure parameters and fabrication recipes. In addition, it enables a reliable definition of fine lateral features due to its lower aspect ratio (low thickness relative to feature width). The exposed regions were removed using a developer (AR 600-546), followed by ion beam etching to open the Cr and AZ 1505 layers. Subsequently, oxygen (O_2_) plasma reactive ion etching (Oxford Instruments, Ionfab 300) was used to remove the electron beam resist. The bottom resist layer was protected by the Cr layer. A 170-nm-thick TiO_2_ layer was then deposited via ALD (Sentech SILAYO). To prepare for planarization, an additional AZ 1505 layer was applied to minimize surface roughness induced by TiO_2_ deposition. The TiO_2_ layer was etched down to the target thickness of 125 nm using ion beam etching, and lastly, the AZ 1505 photoresist was removed via O_2_ plasma cleaning (Oxford Instruments, PlasmaPro 100).

### Preparation of the gain medium

We dissolved 6.2 mg of Rh6G dye in powder form in 2 ml of cyclopentanone and mixed the solution with 1.6 ml of the negative-tone photoresist SU8-2005. Next, we ultrasonicated the solution for 10 min. The concentration of the Rh6G in the final solution is 0.165 wt %. We spin-coated the metasurfaces with this solution for a duration of 5 s with the spin coating speed of 500 rpm, followed by 60 s with the speed of 6000 rpm, which resulted in a final film thickness of 880 nm. Last, we baked the coated samples for 2 min at 95°C to evaporate the excess cyclopentanone. If required, the SU8 film can be removed using acetone after performing measurements on the samples.

### Experimental setup for lasing metasurface characterizations

The sample was pumped using a 532-nm pulsed laser (Teem Photonics, type STG-03E-130) with a pulse width of 0.5 ns and a maximum energy per pulse of 3 μJ. The collimated laser beam illuminated an adjustable square aperture (maximum open aperture 12 mm by 12 mm), which was then imaged onto the selected metasurface, resulting in a homogeneous illumination limited to the metasurface footprint. The emission from the metasurface was collected using a 60×/0.9 numerical aperture (NA; Olympus UPLFLN) objective and then forwarded either to a spectrometer for spectroscopic analysis or to a camera for BFP imaging of emission. A sketch of the setup is illustrated in fig. S4. All the measurements are performed with a repetition rate of 1 Hz and an integration time of 1 s to minimize sample damage caused by bleaching of the Rh6G when performing a sequence of measurements, unless stated otherwise.

#### Power-dependent fluorescence emission spectroscopy

The emission from the sample was collected for a pump pulse energy varied from 0 to 9 nJ and directed to a spectrometer (FLAME-S-VIS-NIR-ES, 1.33-nm FWHM). The pump power was varied using an adjustable neutral density filter located in front of the excitation laser.

#### BFP and momentum-resolved spectroscopy measurements

The Fourier image of the emission was formed at the BFP of the collecting objective [60×/0.9 NA (Olympus UPLFLN)]. A 4f lens system consisting of two 200-mm achromatic doublets (Thorlabs, AC254−200-B) was used for imaging the BFP of the collecting objective onto the sensor of an electron multiplying charge-coupled device camera (Andor iXon Ultra 897). Measurements were performed at different pump pulse energies—below, at, and above the threshold—both with and without band-pass filters at 560 and 570 nm (10-nm FWHM) in the detection path. For the momentum-resolved spectroscopy, we projected the BFP of the collection objective on the entrance slit of an imaging spectrometer (Andor Kymera 328i).

## Supplementary Materials

### This PDF file includes:

Supplementary Text Figs. S1 to S8 Table S1 References
