# Supplementary material for: Holographic lasing with dielectric metasurfaces
Source: Sci Adv. 2026 May 29;12(22):eaea7345. doi: 10.1126/sciadv.aea7345 (PMC13220854; doi:10.1126/sciadv.aea7345)
Supplement: Supplementary file 1 — Supplementary Text Figs. S1 to S8 Table S1 References [file sciadv.aea7345_sm.pdf]

Science Advances
AAAS

Supplementary Materials for

Holographic lasing with dielectric metasurfaces

Ayesheh Bashiri *et al.*

Corresponding author: Ayesheh Bashiri, Ayesheh.bashiri@uni-jena.de

Sci. Adv. **12**, eaea7345 (2026)
DOI: 10.1126/sciadv.aea7345

This PDF file includes:

Supplementary Text
Figs. S1 to S8
Table S1
References

Supplementary Text

Mode Q-factor evaluation

To examine the relationship between band dispersion and cavity quality factor (Q), we analyzed the simulated angle- and wavelength-resolved emission enhancement of the dipole emitters coupled to the designed metasurface laser (relative to the emission from the same emitters in vacuum) below threshold (Fig. S2A). Three angular cross sections through the dispersive band are marked by dashed lines at $\theta = 0°$ (1, blue), $\theta = 0.6°$ (2, green), and $\theta = 1.1°$ (3, orange). Figure S2B shows the corresponding emission enhancement spectra extracted along these cross-sections, with the same color scheme. For each cross-section, the full width at half maximum (FWHM) and the Q factor were obtained from Lorentzian fits of the spectral peaks associated with the same dispersive band. The calculated Q factors demonstrate a clear dependence on the local slope of the band: the highest Q is observed at $\theta = 0°$, where the dispersion is flattest (band edge), followed by $\theta = 0.6°$, and the lowest Q occurs at $\theta = 1.1°$, where the dispersion exhibits the steepest slope.

Linewidth extraction and spectrometer response correction

Linewidth extraction and spectrometer response correction:

To accurately determine the intrinsic lasing mode linewidth, we performed a spectral analysis as follows: (1) Isolation of the cavity mode from the dye fluorescence background, (2) measurement of the instrument line shape (ILS) of the spectrometer under identical acquisition settings, and (3) forward-modeled fitting (*42*), where a Lorentzian function representing the lasing mode is convolved with the measured ILS and fitted to the experimental spectrum. This procedure yields the lasing mode linewidth as a function of pump energy. The spectrometer's instrumental response was experimentally characterized using a narrow 532 nm pulsed laser (the same excitation source and optical configuration used for the metasurface measurements). The intrinsic linewidth of this laser is very small compared to the spectrometer resolution (Teem Photonics STG-03E-130 datasheet: Linewidth: 0.8 pm); therefore, the measured response represents the spectrometer ILS. Under identical spectrometer settings, the measured ILS exhibited a FWHM of ≈1.67 nm, corresponding to a spectral resolution of ≈1.68 nm near 560 nm. Because this resolution is comparable to the apparent linewidth of the lasing peak in the raw spectra (≈1.8 nm), a deconvolution was necessary to recover the lasing mode linewidth.

At and above the lasing threshold, each spectrum was normalized to a mode-free fluorescence reference obtained from the dye-coated substrate. This normalization suppresses the broad fluorescence envelope while preserving the narrow cavity resonance. The isolated peak was then analyzed using forward convolution fitting, in which a Lorentzian function representing the lasing mode was convolved with the experimentally measured ILS and fitted to the measured spectrum to extract the intrinsic linewidth. This approach ensures that the retrieved linewidth accurately reflects the true lasing mode, free from spectrometer-broadening and fluorescence background contributions.

Intensity uniformity analysis

To quantitatively evaluate the uniformity of the holographic image, we followed the definition used in (*33*). The parameter σ_{rms} quantifies the variation of the pixel intensities within a selected holographic region and is defined as $\sigma_{\mathrm{rms}} = \left[\frac{\langle (I(x,y)-\bar{I})^2 \rangle}{\bar{I}^{\,2}}\right]^{\frac{1}{2}}$, where $I(x, y)$ denotes the intensity within that region, $\bar{I} = \langle I(x, y) \rangle$ stands for the average intensity, and $\langle . \rangle$ is the average operator. We selected the letter "S" in both the experimental BFP image (Fig. 3E) and the simulated holographic pattern (Fig. 2E), as this feature is clearly resolved in both cases and serves as a representative example of the proof of concept. The calculated uniformity σ_{rms} values are approximately 58% for the experimental image and 51% for the simulation, demonstrating close quantitative correspondence and confirming that the measured intensity distribution faithfully reproduces the predicted holographic output.

Double-mode lasing metasurface

We examined an additional fabricated metasurface, featuring the same spatial distribution of nanoresonators as the metasurface introduced in the main text, but with slightly different nanoresonator dimensions (see table S1). The evolution of output emission intensity with increasing pump energy (Fig. S6) exhibits multimode lasing behavior, as evidenced by two distinct lasing peaks at around 560 nm and 567 nm, with lasing thresholds of 7.9 nJ and 7.95 nJ, respectively (also see Fig. S8 for the angular-resolved emission spectra). The lasing peak at 560 nm originates from the second-order Bragg diffraction, similar to the previously investigated metasurface (indicated by a white arrow in Fig. 2B). The second lasing peak at 567 nm arises from the upper band edge of the stop-band shown in Fig. 2B with a blue arrow. Interestingly, due to having a different symmetry from that of the normal incidence plane wave, this mode is not excited at normal incidence and thus cannot couple to the far-field in the normal direction (*11*). The evolution of the far-field emission pattern as the pump pulse energy increases from below to above the threshold, both with and without band-pass filters at 560 nm and 570 nm, is illustrated in Fig. S7. While the BFP images shown in Fig. S7, D-F (560 nm) highly resemble those in Fig. 3C-E due to their origin from the same mode, the far-field patterns in Fig. S7, G-I (570 nm) exhibit notable differences. Importantly, we observe a doubling of the "FSU" pattern above the threshold at 570 nm (Fig. S7I). This behavior originates from the momentum-space distribution of the corresponding lasing mode. Unlike the 560 nm lasing mode, which produces a single on-center diffraction peak at $k_{||}= 0$, the 567 nm mode exhibits two dominant lasing diffraction peaks located symmetrically above and below the center of the BFP image. These peaks are the momentum-space distribution of the lasing mode.

The holographic far-field can be interpreted using a standard structure-factor (phased-array) framework (*31*). The spatial arrangement of the nanoresonators acts as a binary amplitude mask whose FT (structure factor) $S(k)$ encodes the "FSU" pattern, while the lasing mode induces the excitation phase and amplitude distribution across the nanoresonators. The experimentally observed far-field distribution is therefore governed by the convolution of the FT of the nanoresonators' arrangement (structure factor) with the momentum-space distribution of the lasing mode. For the 567 nm lasing mode, the momentum-space distribution can be approximated by a double-peaked function $M(k) \approx (k-k_0) + (k+k_0)$, corresponding to the two off-center lasing diffraction peaks observed in the BFP. Consequently, the far-field becomes proportional to $S(k) * M(k)$ (here $*$ denotes convolution), producing two shifted replicas of the hologram and thus the observed doubling of the "FSU" pattern above threshold.

Second-order correlation measurements: Feasibility and limitations

We attempted second-order intensity correlation measurements using a Hanbury Brown–Twiss (HBT) configuration to probe the photon statistics of the metasurface emission. The metasurface laser was pumped at 532 nm using the same excitation conditions as in the lasing experiments (pulse width 0.5 ns, repetition rate 1 Hz). The emission was collected and coupled into a 50:50 fiber beam splitter, and the two outputs were detected by identical single-photon avalanche detectors (Laser Components COUNT Blue series; dead time ≈ 40 ns). Photon arrival times were recorded using a quTAG time-tagging module (time resolution 1 ps). Data were acquired for 30 s with a 100 ps bin width, 1000 bins, and a coincidence delay window of $\pm$40 ns. As we mentioned before, in our lasing characterization, a low repetition rate (1 Hz) and short acquisition time (1 s) were used to minimize photobleaching and irreversible degradation of the Rh6G gain medium, and to maintain stable conditions throughout power-dependent measurements. However, here the acquisition time was increased to 30 s to improve photon statistics. Despite this, the combination of the low repetition rate and the limited integration time required for dye stability results in a very small number of excitation events (≈ 30 pulses per acquisition), leading to insufficient coincidence statistics to construct a reliably normalized $g^{(2)}(\tau)$. In addition, for pulsed excitation, the evaluation of $g^{(2)}(0)$ relies on comparing the coincidence peak at zero delay to side peaks separated by integer multiples of the pulse period. In our case, the pulse period is 1 s, whereas the maximum accessible coincidence window of the correlator in histogram mode is approximately 4.3 ms. Consequently, only the central coincidence peak lies within the measurable temporal window, while the nearest side peaks fall far outside the detection range. Capturing at least one side peak within the available window would require increasing the pump repetition rate to ≥ 250 Hz. Such an increase, however, leads to rapid degradation of the Rh6G gain medium, preventing stable lasing and repeatable measurements.

These constraints severely limit the feasibility of photon-correlation measurements under stable operating conditions for our dye-based metasurface laser. For this reason, we do not present raw coincidence histograms or extracted $g^{(2)}(\tau)$ curves, as they would not provide reliable statistical information. We note that photon-correlation measurements become considerably more feasible in metasurface laser platforms employing more photostable gain media, such as III–V semiconductor systems, where higher repetition rates and longer integration times can be used without compromising gain-material stability.

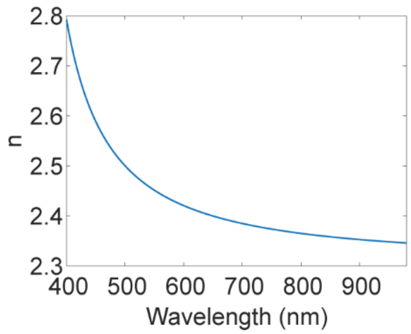

Fig. S1. TiO_2 refractive index information. Ellipsometrically measured real (n) part of the refractive index of TiO_2 thin films used for the nanofabrication of our metasurfaces. The imaginary part of the refractive index is negligible in this spectral range.

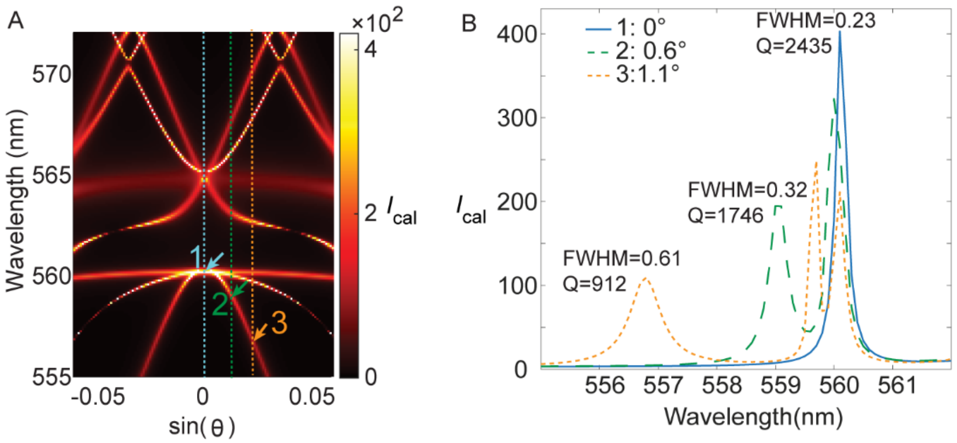

Fig. S2. Mode Q-factor evaluation. (**A**) Simulated angle- and wavelength-resolved emission enhancement of the designed metasurface laser. Dashed lines indicate the three angular cross sections at θ = 0° (1, blue), 0.6° (2, green), and 1.1° (3, orange). I_{cal} stands for average electric field intensity enhancement with respect to normal-incidence plane-wave illumination, defined as $I_{\mathrm{cal}}=\langle\frac{|\mathbf{E}|^2}{|E_0|^2}\rangle$. Here, the average is taken over the SU8 layer containing Rh6G. (**B**) Corresponding emission spectra extracted along the marked cross sections, showing the highest Q factor at the band edge (θ = 0°) where the dispersion is flat, and lower Q factors at larger angles with increasing band slope.

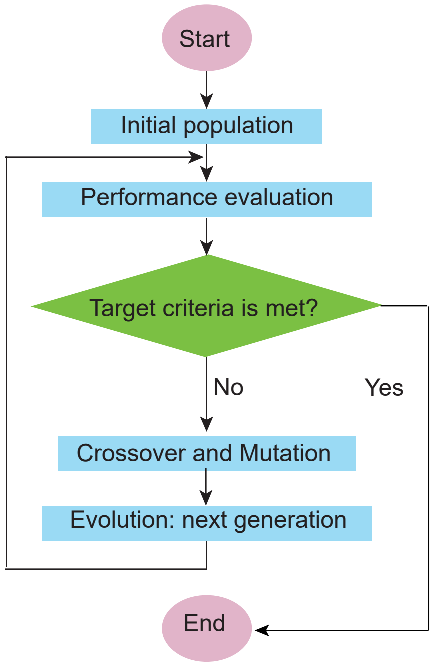

Fig. S3. Optimization process using Genetic algorithm. The flowchart illustrates the iterative process used to optimize the arrangement of nanoresonators in the metasurface, ultimately achieving the desired far-field pattern.

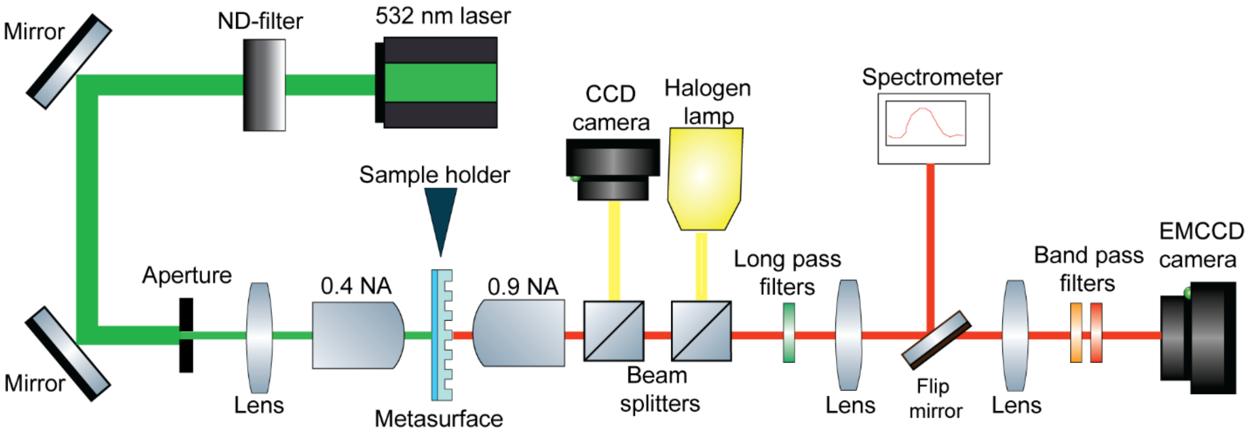

Fig. S4. Lasing characterization setup. The sample is pumped using a 532-nm pulsed laser. The collimated laser beam illuminates an adjustable aperture, which is imaged onto the selected metasurface, resulting in a homogeneous illumination limited to the metasurface footprint. The emission from the metasurface is collected using a 0.9NA objective and then forwarded either to a spectrometer for the spectroscopy measurements or to the EMCCD camera for BFP measurements.

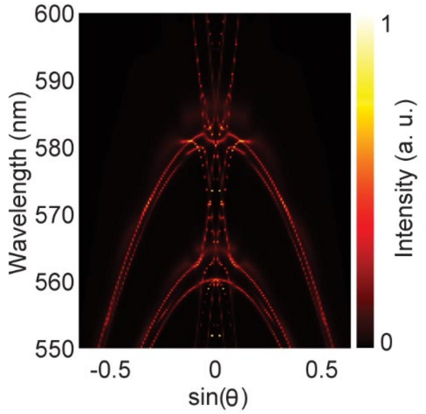

Fig. S5. Simulated angle- and wavelength-resolved emission enhancement. The angle- and wavelength-resolved emission enhancement (normalized to its maximum) is calculated for an angle $\varphi = 0$ and θ up to ± 40 degrees averaged over TE and TM polarization of the incident light.

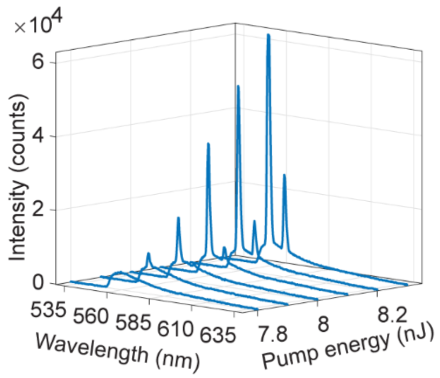

Fig. S6. Double-mode lasing spectra. Evolution of the emission spectra at different pump energies, for a metasurface featuring a slightly larger diameter of the nanoresonators while preserving their spatial arrangement. The occurrence of two sharp peaks indicates multimode lasing behavior.

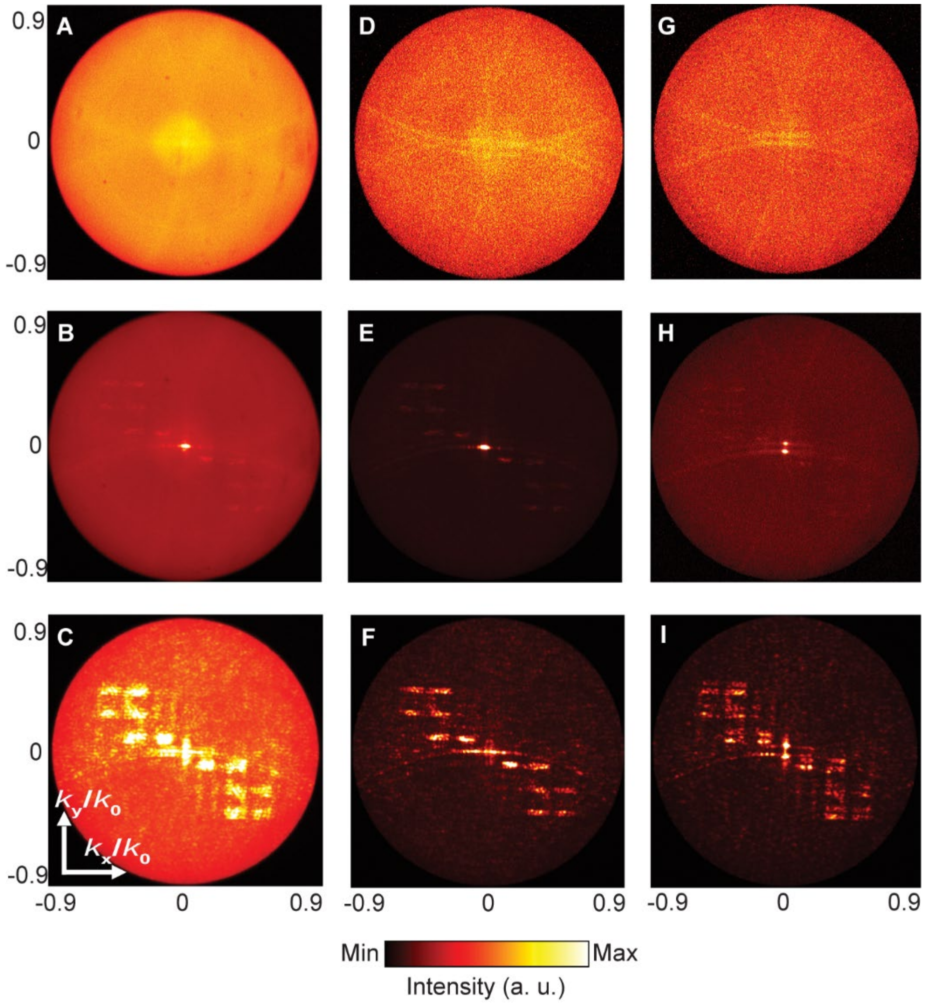

Fig. S7. Double-mode lasing BFP images. (**A**) Below, (**B**) slightly above, (**C**) well above the threshold, and without a band-pass filter. (**D**) Below, (**E**) slightly above, (**F**) well above the threshold, and with a 560 nm band-pass filter. (**G**) Below, (**H**) slightly above, (**I**) well above the threshold, and with a 570 nm band-pass filter. At slightly above threshold (B, E, H), the main lasing peak appears, followed by the emergence of the "FSU" pattern. At well above threshold (C, F, I), while the strong central lasing peak is suppressed using a spatial filter, the lasing target pattern becomes dominant. Additionally, the target pattern aligns with the main lasing mode, appearing as a single pattern at 560 nm (F) and a double pattern at 570 nm (I).

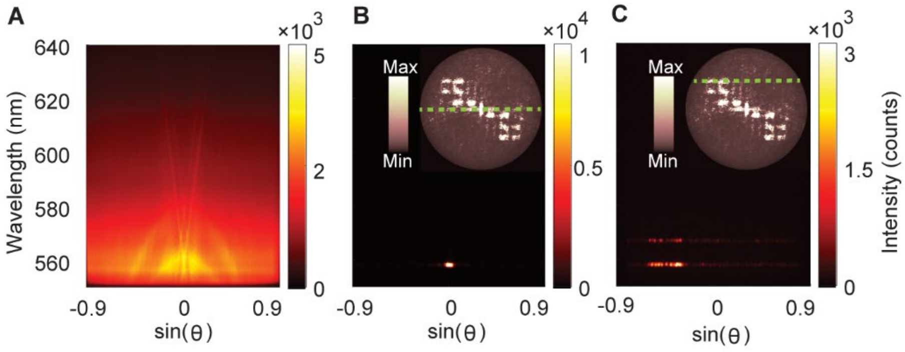

Fig. S8. Double-mode lasing angular-resolved spectra. (**A**) A thin slice ($k_y = 0$) of the BFP image shown in Fig. S7A is spectrally resolved for the pump power below the threshold. Note that for better visibility, the measurement below the threshold is performed with an excitation laser repetition rate of 4 kHz and integrated for 30 s. (**B**) Above threshold, similar to (A), as shown in the inset with a dashed green line, the slice is cut from the center of the BFP image. (**C**) Well above the threshold, a cross-section through the upper part of "FSU" (see inset) is spectrally resolved, revealing the double-mode lasing at 560 nm and 570 nm. Note, the second peak is absent in (B) as it appears slightly above and below the $\theta = 0$, while the horizontal cut is taken from the center (dashed green line). The spatial filter is removed for the momentum-resolved spectroscopy.

Table S1. Dimensions of the designed and both fabricated metasurfaces, showing single and double peak lasing.

Metasurface parameters	Nanodisk diameter (nm)	Nanodisk height (nm)	Period (nm)
Designed	170	125	365
Fabricated (single-mode)	170	120	365
Fabricated (double-mode)	175	120	365

REFERENCES

1. N. Yu, F. Capasso, Planar photonics with metasurfaces. *Science* **339**, 6125 (2013).

2. A. M. Shaltout, V. M. Shalaev, M. L. Brongersma, Spatiotemporal light control with active metasurfaces. *Science* **364**, 6441 (2019).

3. A. H. Dorrah, F. Capasso, Tunable structured light with flat optics. *Science* **376**, 6591 (2022).

4. A. Arbabi, Y. Horie, M. Bagheri, A. Faraon, Dielectric metasurfaces for complete control of phase and polarization with subwavelength spatial resolution and high transmission. *Nat. Nanotechnol.* **10**, 937–943 (2015).

5. A. Vaskin, R. Kolkowski, A. F. Koenderink, I. Staude, Light-emitting metasurfaces. *Nanophotonics* **8**, 1151–1198 (2018).

6. S. Liu, A. Vaskin, S. Addamane, B. Leung, M.-C. Tsai, Y. Yuanmu, P. P. Vabishchevich, G. A. Keeler, G. Wang, X. He, Y. Kim, N. F. Hartmann, H. Htoon, S. K. Doorn, M. Zilk, T. Pertsch, G. Balakrishnan, M. B. Sinclair, I. Staude, I. Brener, Light-emitting metasurfaces: Simultaneous control of spontaneous emission and far-field radiation. *Nano Lett.* **11**, 6906–6914 (2018).

7. Y. Mohtashami, R. A. DeCrescent, L. K. Heki, P. P. Iyer, N. A. Butakov, M. S. Wong, A. Alhassan, W. J. Mitchell, S. Nakamura, S. P. DenBaars, J. A. Schuller, Light-emitting metalenses and meta-axicons for focusing and beaming of spontaneous emission. *Nat. Commun.* **12**, 3591 (2021).

8. A. Bashiri, A. Vaskin, K. Tanaka, M. Steinert, T. Pertsch, I. Staude, Color routing of the emission from magnetic and electric dipole transitions of Eu^{3+} by broken-symmetry TiO_2 metasurfaces. *ACS Nano* **18**, 506–514 (2024).

9. A. H. Schokker, A. F. Koenderink, Lasing at the band edges of plasmonic lattices. *Phys. Rev. B* **90**, 15 (2014).

10. S. T. Ha, Y. H. Fu, N. K. Emani, Z. Pan, R. M. Bakker, R. Paniagua-Domínguez, A. I. Kuznetsov, Directional lasing in resonant semiconductor nanoantenna arrays. *Nat. Nanotechnol.* **13**, 1042–1047 (2018).

11. M. Wu, L. Ding, R. P. Sabatini, L. K. Sagar, G. Bappi, R. Paniagua-Domínguez, E. H. Sargent, A. I. Kuznetsov, Bound state in the continuum in nanoantenna-coupled slab waveguide enables low-threshold quantum-dot lasing. *Nano Lett.* **21**, 9754–9760 (2021).

12. S. I. Azzam, K. Chaudhuri, A. Lagutchev, Z. Jacob, Y. L. Kim, V. M. Shalaev, A. Boltasseva, A. V. Kildishev, Single and multi-mode directional lasing from arrays of dielectric nanoresonators. *Laser Photonics Rev.* **15**, 3 (2021).

13. C. Spägele, M. Tamagnone, D. Kazakov, M. Ossiander, M. Piccardo, F. Capasso, Multifunctional wide-angle optics and lasing based on supercell metasurfaces. *Nat. Commun.* **12**, 3787 (2021).

14. Y.-Y. Xie, P.-N. Ni, Q.-H. Wang, Q. Kan, G. Brière, P.-P. Chen, Z.-Z. Zhao, A. Delga, H.-R. Ren, H.-D. Chen, C. Xu, P. Genevet, Metasurface-integrated vertical cavity surface-emitting lasers for programmable directional lasing emissions. *Nat. Nanotechnol.* **15**, 125–130 (2020).

15. P. Fu, P.-N. Ni, B. Wu, X.-Z. Pei, Q.-H. Wang, P.-P. Chen, C. Xu, Q. Kan, W.-G. Chu, Y.-Y. Xie, Metasurface enabled on-chip generation and manipulation of vector beams from vertical cavity surface-emitting lasers. *Adv. Mater.* **35**, 12 (2023).

16. Y. Zeng, X. Sha, C. Zhang, Y. Zhang, H. Deng, H. Lu, G. Qu, S. Xiao, S. Yu, Y. Kivshar, Q. Song, Metalasers with arbitrarily shaped wavefront. *Nature* **643**, 1240–1245 (2025).

17. F. Laux, N. Bonod, D. Gérard, Single emitter fluorescence enhancement with surface lattice resonances. *J. Phys. Chem. C* **121**, 13280–13289 (2017).

18. G. Cáceres-Aravena, R. A. Vicencio, Perfect localization on flat-band binary one-dimensional photonic lattices. *Phys. Rev. A* **100**, 013803 (2019).

19. A. Krasnok, S. Glybovski, M. Petrov, S. Makarov, R. Savelev, P. Belov, C. Simovski, Y. Kivshar, Demonstration of the enhanced Purcell factor in all-dielectric structures. *Appl. Phys. Lett.* **108**, 211105 (2016).

20. K. Sun, K. Wang, W. Wang, Y. Cai, L. Huang, A. Alù, Z. Han, High-Q photonic flat-band resonances for enhancing third-harmonic generation in all-dielectric metasurfaces. *Newton* **1**, 100057 (2025).

21. A. I. Kuznetsov, A. E. Miroshnichenko, M. L. Brongersma, Y. S. Kivshar, B. Luk’yanchuk, Optically resonant dielectric nanostructures. *Science* **354**, 6314 (2016).

22. I. Staude, J. Schilling, Metamaterial-inspired silicon nanophotonics. *Nat. Photonics* **11**, 274–284 (2017).

23. B. Evlyukhin, S. M. Novikov, U. Zywietz, R. L. Eriksen, C. Reinhardt, S. I. Bozhevolnyi, B. N. Chichkov, Demonstration of magnetic dipole resonances of dielectric nanospheres in the visible region. *Nano Lett.* **12**, 3749–3755 (2012).

24. W. L. Vos, R. Sprik, A. van Blaaderen, A. Imhof, A. Lagendijk, G. H. Wegdam, Strong effects of photonic band structures on the diffraction of colloidal crystals. *Phys. Rev. B* **53**, 16231–16235 (1996).

25. C. M. Soukoulis, Ed., *Photonic Crystals and Light Localization in the 21st Century* (Springer Science & Business Media, 2012).

26. A. David, H. Benisty, C. Weisbuch, Photonic crystal light-emitting sources. *Rep. Prog. Phys.* **75**, 126501 (2012).

27. C. C. Liu, H. H. Hsiao, Y. C. Chang, Nonlinear two-photon pumped vortex lasing based on quasi-bound states in the continuum from perovskite metasurface. *Sci. Adv.* **9**, eadf6649 (2023).

28. H. Xue, J. Niu, C. Wang, S. Chen, C. Lu, P. Zhang, F. Jin, C. Xie, L. Shi, Lasing action assisted by bound states in the continuum in a silicon metasurface. *Opt. Laser Technol.* **177**, 111103 (2024).

29. M. Wu, S. T. Ha, S. Shendre, E. G. Durmusoglu, W.-K. Koh, D. R. Abujetas, J. A. Sánchez-Gil, R. Paniagua-Domínguez, H. V. Demir, A. I. Kuznetsov, Room-temperature lasing in colloidal nanoplatelets via Mie-resonant bound states in the continuum. *Nano Lett.* **20**, 6005–6011 (2020).

30. A. H. Schokker, A. F. Koenderink, Statistics of randomized plasmonic lattice lasers. *ACS Photonics* **2**, 1289–1297 (2015).

31. A. H. Schokker, A. F. Koenderink, Lasing in quasi-periodic and aperiodic plasmon lattices. *Optica* **3**, 686–693 (2016).

32. G. Zheng, H. Mühlenbernd, M. Kenney, G. Li, T. Zentgraf, S. Zhang, Metasurface holograms reaching 80% efficiency. *Nat. Nanotechnol.* **10**, 308–312 (2015).

33. K. Huang, H. Liu, F. J. Garcia-Vidal, M. Hong, B. Luk'yanchuk, J. Teng, C.-W. Qiu, Ultrahigh-capacity non-periodic photon sieves operating in visible light. *Nat. Commun.* **6**, 7059 (2015).

34. M. Gen, R. Cheng, *Genetic Algorithms and Engineering Design* (Wiley, 1996), 10.1002/9780470172254.

35. L. Langguth, A. H. Schokker, K. Guo, A. F. Koenderink, Plasmonic phase-gradient metasurface for spontaneous emission control. *Phys. Rev. B* **92**, 205401 (2015).

36. Z. Wang, S. Wan, Y. Shi, Z. Li, Vectorial fluorescence control with light-emitting metasurfaces for unidirectional emission and incoherent holography. *Adv. Mater.* **37**, e2502682 (2025).

37. Y. Baum, J. Scheuer, Meta-holograms under incoherent illumination: Image properties and spackle pattern. *Opt. Express* **31**, 36981 (2023), 36991.

38. R. Zhu, T. Qiu, J. Wang, S. Sui, C. Hao, T. Liu, Y. Li, M. Feng, A. Zhang, C.-W. Qiu, S. Qu, Phase-to-pattern inverse design paradigm for fast realization of functional metasurfaces via transfer learning. *Nat. Commun.* **12**, 2328 (2021).

39. Z. Li, R. Pestourie, Z. Lin, S. G. Johnson, F. Capasso, Empowering metasurfaces with inverse design: Principles and applications. *ACS Photonics* **9**, 2178–2192 (2022).

40. A. C. Overvig, S. Shrestha, S. C. Malek, M. Lu, A. Stein, C. Zheng, N. Yu, Dielectric metasurfaces for complete and independent control of the optical amplitude and phase. *Light Sci. Appl.* **8**, 92 (2019).

41. A. Tripathi, H.-R. Kim, P. Tonkaev, S.-J. Lee, S. V. Makarov, S. S. Kruk, M. V. Rybin, H.-G. Park, Y. Kivshar, Lasing action from anapole metasurfaces. *Nano Lett.* **21**, 6563–6568 (2021).

42. R. C. Aster, B. Borchers, C. H. Thurber, *Parameter Estimation and Inverse Problems* (Academic, ed. 3, 2018).
